# Atypical antioxidant activity of non-phenolic amino-coumarins†

**DOI:** 10.1039/c7ra12000a

**Published:** 2018-01-09

**Authors:** Daniel Zúñiga-Núñez, Pablo Barrias, Gloria Cárdenas-Jirón, M. Soledad Ureta-Zañartu, Camilo Lopez-Alarcón, F. Eduardo Morán Vieyra, Claudio D. Borsarelli, Emilio I. Alarcon, Alexis Aspée

**Affiliations:** Facultad de Química y Biología, Universidad de Santiago de Chile (USACH) Casilla 40, Correo 33 Santiago Chile alexis.aspee@usach.cl; Departamento de Química Física, Facultad de Química, Pontificia Universidad Católica de Chile Av. Vicuña Mackenna 4860 Santiago Macul Chile; Instituto de Bionanotecnología del NOA (INBIONATEC), Universidad Nacional de Santiago del Estero (UNSE) CONICET. RN9, km 1125 CP4206 Santiago del Estero Argentina; Bio-nanomaterials Chemistry and Engineering Laboratory, Division of Cardiac Surgery, University of Ottawa Heart Institute 40 Ruskin St. Ottawa Ontario K1Y 4W7 Canada; Department of Biochemistry, Microbiology, and Immunology, Faculty of Medicine, University of Ottawa 451 Smyth Road K1H 8M5 Ottawa ON Canada

## Abstract

Coumarin compounds have been described as anti-inflammatories, and chemotherapeutic agents as well as antioxidants. However, the origin of the antioxidant activity of non phenolic coumarins remains obscure. In the present report, we demonstrate that non-phenolic 7-dialkyl-aminocoumarins may also have significant antioxidant properties against free radicals derived from 2,2′-azobis(2-amidinopropane) dihydrochloride under aerobic conditions. This atypical behaviour is due to the presence of traces of very reactive hydroxycinnamic acid-type compounds. Changing functional groups at the C-3 and C-4 positions shifts the reactivity of the compounds from peroxyl to alkoxyl free radicals. Kinetic and theoretical studies based on Density Functional Theory support the formation of reactive hydroxycinnamic acid and directly link the antioxidant behaviour of the compounds to hydrogen atom transfer.

## Abstract 2

Relevant antioxidant properties of non-phenolic 7-dialkyl-aminocoumarins against free radicals derived from 2,2′-azobis(2-amidinopropane) dihydrochloride under aerobic conditions have been experimentally and theoretically demonstrated.

## Graphical abstract

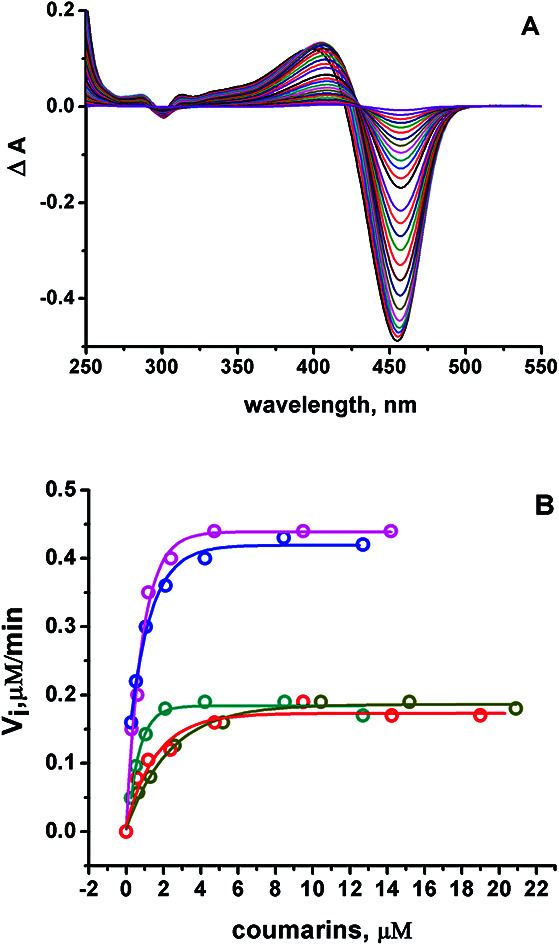

## 1. Introduction

Coumarins, 1,2-benzopyrone compounds, have been employed for labelling of proteins and DNA,^1^ design of fluorescent probes,^2^ as photoactive components in solar cells,^3^ and as laser dyes.^4,5^ In a pharmacological context, several coumarins have anti-inflammatory effects,^6^ and some display chemotherapeutic properties.^6–8^ In addition, hydroxycoumarin compounds have shown significant scavenging antioxidant capacity towards (2,2-diphenyl-1-picrylhydrazyl) radicals (DPPH) and peroxyl radicals derived from 2,2′-azobis-(2-amidinopropane)hydrochloride (AAPH) thermolysis.^9–12^ In particular, peroxyl radicals–coumarin reactions occur *via* electron transfer (ET) and hydrogen atom transfer (HAT) from the phenol coumarin group.^13–15^ Some studies have also reported antioxidant properties for coumarins that have no labile hydrogen atoms in their structure, which suggests a much more complex oxidation mechanism.^16–18^

**Scheme 1:**
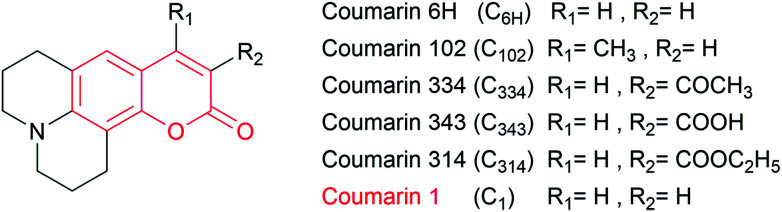
Structure of coumarin and 7-dialkyl-aminocoumarins.

In the present work, we studied the reaction of several 7-dialkyl-aminocoumarins without a phenolic OH moiety (7ACs, Scheme 1) with AAPH-derived free radicals by spectroscopy and UHPLC MS/MS chromatography. Specifically, the kinetic evaluation of the reaction of 7ACs and peroxyl radicals was studied by a combination of experimental and density functional theory (DFT) calculations.

## 2. Experimental section

### 2.1 Materials

Coumarin (C_1_), coumarin 6H (C_6H_), coumarin 102 (C_102_), coumarin 153 (C_153_), coumarin 334 (C_334_), coumarin 343 (C_343_), coumarin 314 (C_314_), NaHPO_4_·H_2_O (p.a), Na_2_HPO_4_ (p.a), 2,2′-azobis (2-amidinopropane)dihydrochloride (AAPH), and methanol (HPLC grade) were purchased from Sigma-Aldrich. NaOH (p.a) and Na_3_C_6_H_5_O_7_ (p.a) were obtained from Merck.

### 2.2 Sample preparation

All solutions were prepared by addition of small aliquots of concentrated 7ACs methanol stock solutions into phosphate buffer (20 mM, pH 7.0). The concentration of coumarins in the methanol stock were determined using the extinction coefficients in methanol: C_1_*ε* = 10 500 M^−1^ cm^−1^ at 278 nm, C_314_*ε* = 47 000 M^−1^ cm^−1^ at 436 nm, C_102_*ε* = 21 500 M^−1^ cm^−1^ at 389 nm, C_6H_*ε* = 25 000 M^−1^ cm^−1^ at 396 nm, C_343_*ε* = 44 300 M^−1^ cm^−1^ at 430 nm, and C_334_*ε* = 47 300 M^−1^ cm^−1^ at 450 nm.^19^ The extinction coefficient was determined in water for calculate concentration in the aqueous solutions. C_1_*ε* = 10 700 M^−1^ cm^−1^ at 278 nm, C_314_*ε* = 42 260 M^−1^ cm^−1^ at 448 nm, C_102_*ε* = 18 690 M^−1^ cm^−1^ at 395 nm, C_6H_*ε* = 21 260 M^−1^ cm^−1^ at 402 nm, C_343_*ε* = 47 260 M^−1^ cm^−1^ at 437 nm, and C_334_*ε* = 47 170 M^−1^ cm^−1^ at 460 nm. It is important to note that, the concentration of methanol in all the samples were always lower than 0.3% v/v. Phosphate buffer, AAPH and NaOH solutions were prepared using Milli-Q water (18.2 μS).

### 2.3 Consumption of dyes elicited by peroxyl radicals

The kinetics of consumption of 7ACs by peroxyl radicals were evaluated from the decrease of the absorbance band (UV-visible Agilent 8453 Spectrophotometer) as a function of the incubation time in 10 mM AAPH at 37 °C under aerobic conditions. Estimation of the coumarin consumption at low concentration was carried out by fluorescence measurements in a Shimadzu RF-5301 PC spectrofluorometer. The consumption of coumarins was followed by the decrease of the fluorescence of each coumarin as a function of the AAPH incubation time. Consumption measurements for the highest coumarin concentrations were followed by absorbance measurements, at 20 nm longer than the maximum for avoiding any interference of product formed. That wavelength limit was selected after analysis of the multivariate curve resolution.

In addition, measurements of UHPLC MS/MS were carried out for quantifying the direct consumption of 7ACs elicited by AAPH incubation, using an UHPLC Ultimate 3000 RSLC coupled with a LTQ XL linear Ion Trap Mass Spectrometer (Thermo scientific). Briefly, aliquots from the reaction media were taken at different incubation times (0, 10, 20, 30, 40, 50 and 60 min), and diluted in 0.1% formic acid until 1 μg mL^−1^ and immediately injected into UHPLC-MS/MS employing a Hypersil GOLD C8 column (50 × 4.6 mm, 1.9 μm, Thermo Fisher Scientific). An isocratic mobile phase (30/70) 0.1% formic acid/methanol (0.1% formic acid) at 0.4 mL min^−1^ was employed, and monitored [M + H]^+^ and mass fragmentation of the main ion mass products in full scan mode (100 to 700 *m*/*z*).

### 2.4 Kinetic of hydrolysis on basic media

The hydrolysis of 7-ACs was measured in 1 M NaOH following by spectroscopic changes on the absorbance spectra on time. All measurements were carried out at room temperature.

### 2.5 Electrochemical assessment of 7-ACs

Electrochemical measurements were performed with an Autolab (Eco Chemie, The Netherlands, electrochemical work station) using a three-compartments cell with a platinum spiral of large area as counter electrode, an Ag/AgCl/saturated KCl as reference electrode and glassy carbon as working electrode. Oxidation potential of coumarins measured for all coumarins at 0.1 V s^−1^ in 100 mM phosphate buffer pH 7 and 1 M NaOH.

### 2.6 Theoretical calculations

All the ground state calculations were performed using density functional theory (DFT) with the Gaussian 09 package.^20^ Stationary points on the potential energy surface were obtained using the B3LYP hybrid density functional method.^21,22^ The molecular geometry optimization was performed in the gas phase using the 6-31G(d,p) basis set for all atoms.^23^ Open-shell species were calculated using the spin-unrestricted formalism with a spin multiplicity of doublet. The converged wave functions were verified by analytical computations of harmonic vibrational frequencies. Gibbs free energies in the gas phase were computed for each species within the ideal gas model, rigid rotor, and harmonic oscillator approximations at a pressure of 1 atm and a temperature of 298.15 K.^24^ The atomic spin densities were evaluated using the natural population analysis (NPA).^25^ The local reactivity criteria at atomic level were calculated using the Fukui function, at every atom for estimating hydrolysis among the different coumarins. A qualitative analysis of the surface diagrams of the Fukui+ evaluated for electrophilic sites in the molecules considered the electronic density of the Lowest Unoccupied Molecular Orbital (LUMO).^26,27^ The bond dissociation energy (BDE) was calculated by means of the homolytic cleavage of hydrogen bond for hydrolysed coumarin and for three hydrogen atoms in coumarins. The energies were obtained from the difference between the neutral species and the free radical species corresponding to the homolytic cleavage of the hydrogen bond.^28^ The correction for the basis set superposition error was not considered here. All the single-point calculations were performed in water using a conductor-like polarizable continuum model (C-PCM) with the standard parameters for water.^29^

## 3. Results and discussion

### 3.1 Evaluation of the free radical reaction of 7ACs with peroxyl radicals

**Scheme 2:**
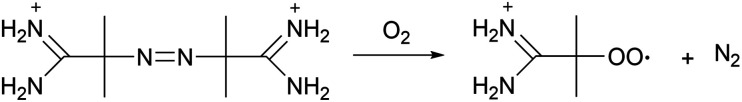
Peroxyl radicals derived from AAPH thermal decomposition.

The assessment of the antioxidant properties of 7ACs was carried out employing a peroxyl radical model with AAPH as a thermal source of free radicals (Scheme 2). Under aerobic conditions; peroxyl radicals rate formation is 0.8 μM min^−1^ at 10 mM AAPH solution at 37 °C.^30^

**Fig. 1:**
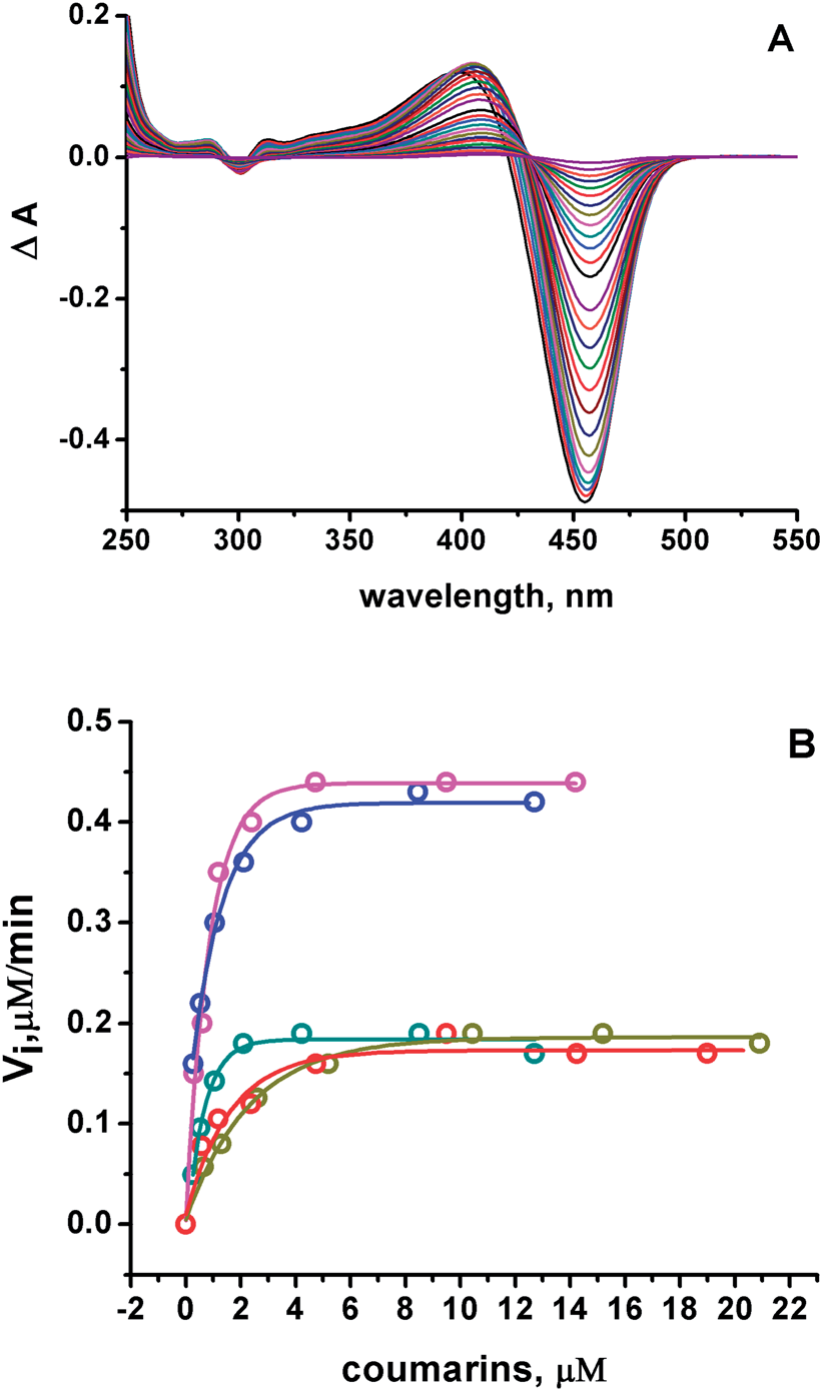
(A) Differential on the absorbance spectra Δ*A* after 15 μM C_314_ incubation with 10 mM AAPH at 37 °C, pH 7.0. (B) Initial consumption rates as a function of the initial 7ACs concentration. 7ACs: C_6H_ (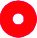), C_334_ (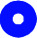), C_343_ (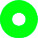), C_314_ (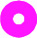), and C_102_ (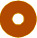). Kinetic rates estimated from absorbance measurements, and fluorescence at low concentrations.

Under these conditions, it was observed a rapid change in the absorbance spectra of all the 7ACs after incubation with AAPH. In Fig. 1A, it is shown as a typical case, the delta absorbance spectra of C_314_ at different incubation times. All the other compounds under study showed similar behaviour (ESI, Fig. S1†). Interestingly, in the case of C_314_ it can be identified different isosbestic points as the progression of the reaction time, suggesting a fast reaction with peroxyl radicals but also a further reaction of the initial oxidation product. Multivariate Curve Resolution using Alternating Least Squares (MCR-ALS) analysis of the UV-Vis spectral changes for C_6H_, C_102_ and C_343_ give satisfactory fits (*e.g. r*^2^ ≈ 1 and lack of fit % LOF < 1)^31^ with the simplest model of two component: kinetic profiles for the coumarin (*R*) decay in concomitant with the growth of oxidation product (P) (Table S1, Fig. S2†). Moreover, it was required an additional extra component associated with an intermediate species on the MCR-ALS analysis for the reaction of both C_334_ and C_314_ to decrease % LOF with acceptable *r*^2^ values (Table S1†).

The evaluation of 7ACs reaction towards peroxyl radicals was estimated considering initial rates from monitoring coumarin bleaching (Fig. 1B). This treatment permitted us to establish a reactivity criteria based on the concentration required for changing from first order reaction to zero order reaction on 7AC concentration, when all generated peroxyl radicals are efficiently trapped as is depicted in eqn (1)–(3).^32,33^1
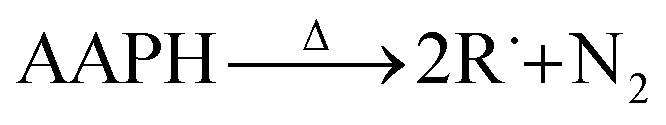

2 R˙ + O_2_ → ROO˙

3 ROO˙ + 7AC → ROOH + 7AC˙

The concentration range of changing order for 7-ACs was lower than 5 μM establishing these compounds as excellent free radical scavengers similar to phenolic compounds such as cinnamic acids.^34–36^ Based on these results, we estimated at high coumarin concentration (zero order) that 2 peroxyl radicals were trapped per C_314_ and C_334_ (Table 1). These results indicate that the stoichiometric peroxyl radical consumption (*n*) is similar to that of reactive phenols or even polyphenols like quercetin.^37^ The same conclusion was drawn from measuring the consumption of 7ACs by UHPLC MS/MS (Fig. S3†). For C_6H_, C_102_ and C_343_*n* values were found between 4 and 5, which would suggest the participation of alkoxyl free radicals as a consequence of a lower reactivity of these coumarins, as shown in eqn (4) and (5).^35^

4 ROO˙ + ROO˙ → 2RO˙ + O_2_

5 RO˙ + 7AC → ROH + 7AC˙

**Table 1:** Calculated Bond Dissociation Energy (BDE) for hydrolysed form (HA, eqn (6)) and parent coumarin (Scheme 3), oxidation potential measured at pH 7 (a) and after hydrolysis in 1 M NaOH (b), and number of peroxyl radicals trapped by coumarin (*n*)

	Calc.				Exp.		*n* b
	BDE (kcal mol^−1^)				Potential/V SHE		
	C_H_O–H	C–H_1_	C–H_2_	C–H_3_, C–H_4_^a^	(a)	(b)	
C_1_	94.1	—	126.4	118.4, 121.8^a^	0.92^c^	0.26	0
C_6H_	87.9	93.8	119.0	117.8	1.18	0.28	3.9 (4.0)
C_102_	88.5	93.9	117.9	121.4	1.17	0.35	4.6 (4.3)
C_334_	82.7	92.5	118.7	119.1^a^	1.24	0.38	2.2 (2.0)
C_343_	87.9	92.5	119.4	119.4	1.28	0.4	5.3 (4.8)
C_314_	81.6	92.8	119.1	119.6	1.26	0.47	2.2 (1.9)

a H_4_ only coumarin C_1_ and C_102_.

b Values between parenthesis were calculated from UHPLC MS/MS.

c Ref. 39.

Thus, in the absence of reactive substrates, self-reaction of peroxyl radicals renders alkoxyl radicals; hence a larger *n* value calculated only reflects a reduced fraction of alkoxyl radical formation rather than peroxyl radicals.^35,38^ It is important to note that an increase in the stoichiometry could also be related to a secondary reaction of peroxyl radicals toward unsaturated double bonds but is questionable considering the low reactivity of peroxyl radicals and the coumarin structures under study.

Despite the high reactivity observed for these 7ACs, large values of oxidation potential measured by cyclic voltammetry (>1.17 volts *vs.* SHE) indicate that electron transfer reactions from 7ACs towards peroxyl radicals, or alkoxy radicals, is improbable (*vide infra*, Table 1). Thus, we considered exploring other plausible routes to explaining such anomalous antioxidant activity.

It is well known that hydrolysis of coumarins at high pH produce a hydroxycinnamic derivative acid, whereas lactonization of these species is observed at low pH.^40,41^ We propose that the antioxidant properties are related to the participation of a small amount of hydroxycinnamic acid type compound present as a consequence of an equilibrium between 7ACs and its hydrolysed form (HA, eqn (6)) at neutral pH. If that were the case, the kinetic of the reaction would be dependent on the amount of the hydroxycinnamic acid compound and its reactivity toward peroxyl radicals. Therefore, HA consumption by free radical reaction would shift the 7AC equilibrium; acting as a reservoir for the HA species considering the slow peroxyl radical flow under our experimental conditions.6
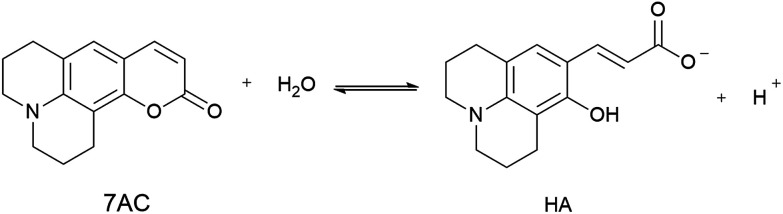


**Fig. 2:**
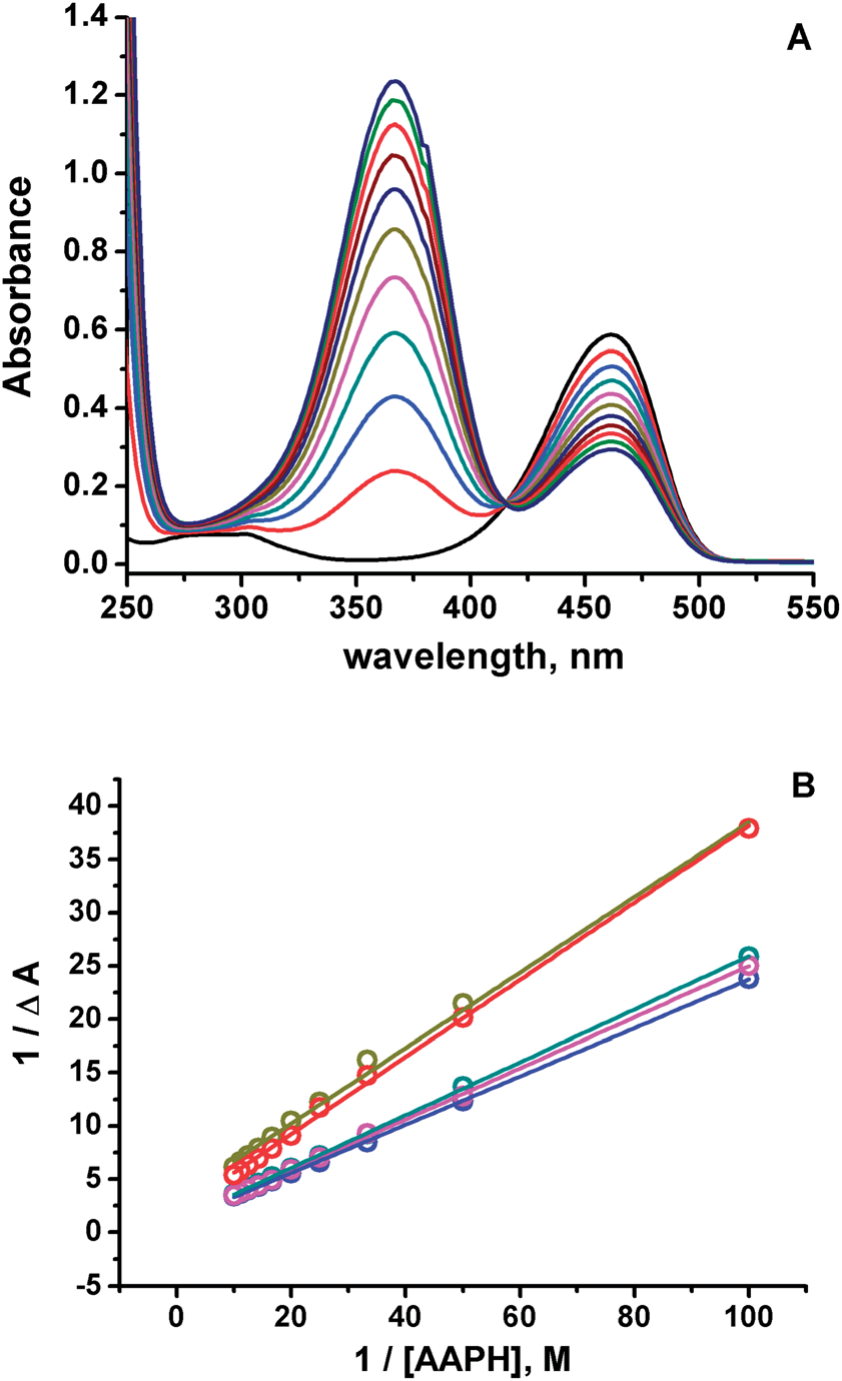
(A) UV-visible absorbance spectra of 15 μM C_334_ measured at different AAPH concentration at 4 °C. (B) Decrease on the 7ACs absorbance with AAPH concentration evaluated at low temperatures, 7ACs: C_6H_ (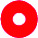), C_334_ (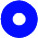), C_343_ (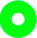), C_314_ (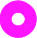), and C_102_ (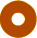).

Spectroscopic evidence for HA species present at neutral pH were obtained from the modification of the 7ACs UV-visible spectrum with AAPH concentration at low temperature (Fig. 2A). Under these conditions there is not relevant AAPH thermal decomposition allowing to establish that 7AC absorbance bleaching with AAPH is due to formation of a complex between HA with AAPH (eqn (7), Fig. 2B). We propose that such HA–AAPH complex is promoted by an electrostatic interaction between negatively charged HA species and cationic AAPH in aqueous solution at neutral pH. In fact, this kind of complex has been previously proposed for pyranine, a negative charged dye.^42^ Consequently, additional experiments were carried out with *o*-coumaric acid as a model for the HA compound that also showed the formation of a complex with AAPH (Fig. S4†).

7 HA + AAPH = [HA − AAPH]

**Scheme 3:**
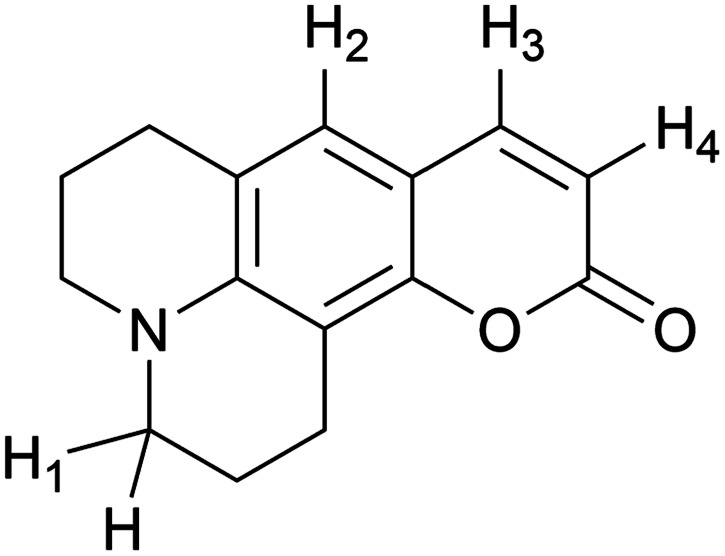
Coumarin hydrogen numbering on calculating BDE in Table 1.

The presence of HA at neutral pH is also in agreement with local reactivity criteria calculated using the Fukui function for nucleophilic attack (*f*+), which predicted that carbon at position 2 is the most electrophilic atom on 7ACs for being attacked during hydrolysis (see Fig. S5, † for all 7ACs). Consequently, evaluation of the hydrolysis under strong basic condition leads directly to formation of the phenolate species of the HA (Fig. S6 and S7†). The difference on the apparent rate constant for the 7ACs suggested dissimilarity on the hydrolysis dependent on the structure of the 7ACs. In fact, it is observed lower hydrolysis rates for all 7ACs in comparison of C_1_ that is in agreement with a resonance effect of the nitrogen moiety decreasing the electrophilicity of the carbonyl group at position 2 (Scheme 3). In addition, inductive and electronic effect of the substituents on C_102_ or C_334_ also reduces the hydrolysis rates compared to C_6H_. In particular, for C_334_, it is observed that the fast hydrolysis reaction reaches equilibrium, whereas the kinetic profile for C_314_ is influenced by an initial hydrolysis of the ethyl ester group for rendering C_343_. In spite of that, there is no a simple relation between the hydrolysis parameters with the reactivity observed of 7ACs against AAPH derived free radicals. Hence, the presence of the nitrogen on 7ACs may play an important role on the redox potential or hydrogen bond energies of the HA species.

### 3.2 Cyclic voltammetry of 7-ACs

To obtain further insight into the oxidation of 7ACs, cyclic voltammetry studies were performed. In particular, irreversible electrochemical oxidation behaviour was observed for all compounds, presenting at neutral pH higher oxidation potentials than C_1_, ranging from 1.9 to 1.3 volts against SHE (Table 1). These results indicate that the oxidation of 7ACs induced by free radicals is not conducted by an electron transfer mechanism. On the other hand, lower oxidation potentials were determined for hydrolysed coumarins at high pH (1 M NaOH) that are associated with the oxidation of deprotonated HA species (phenolate species, A^−^). That could suggests a more favourable redox properties of the HA species at pH 7.0. Consistently, an experimental oxidation potential of 0.7 volts against SHE has been reported for *o*-hydroxycinnamic acid at neutral pH.^43^ These results verify that the reactivity of such coumarin derivatives is expected from the hydrolysed form, HA (Table 1).

### 3.3 Bond dissociation energy of 7-ACs and hydroxycoumarin

The bond dissociation energy (BDE) calculations for the phenolic group (C_H_O–H) for each hydrolysed coumarin showed similar values to hydroxycinnamic acids, such as *o*-hydroxycinnamic acid and coumaric acids.^43^ However, the presence of the amino group on HA would diminish the BDE value respect to the hydrolysed C_1_. Moreover, these dissociation energies are smaller than the dissociation energies of any hydrogen present in the cyclized form of each coumarin (see Table 1, Scheme 3).

**Fig. 3:**
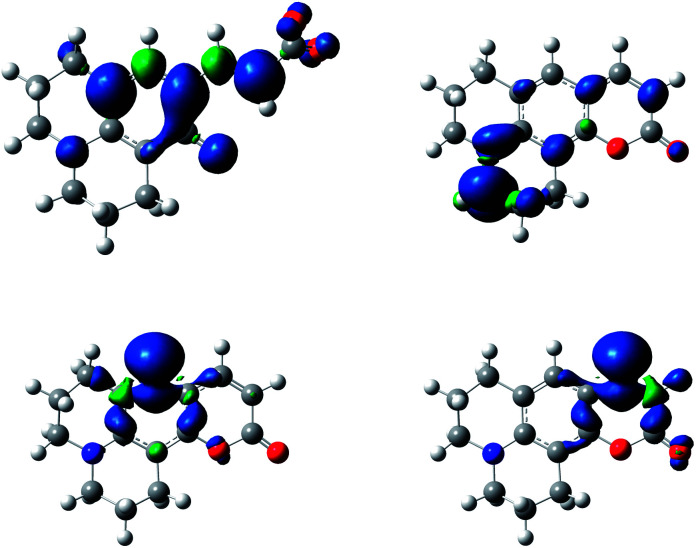
Spin density of C_6H_ hydrolysed free radical (top, left) and spin density of coumarin 6H cycle form by free radical of abstraction of H_1_ (top, right), H_2_ (bottom, left) and H_3_ (bottom, right) at an isosurface value of 0.002, computed using the B3LYP/6-31G(d,p) level of theory and a conductor-like polarizable continuum model (C-PCM) with the standard parameters for water.

The difference in energy between hydrogen atoms in Table 1 reflects the stability of the radical formed once the basic attack occurs. Specifically, spin density calculation presented a greater delocalization in the entire molecule by hydrogen abstraction in the C_H_O–H (hydroxycinnamic acid derivatives, HA), than the spin density of the radicals formed by the abstraction of each of the hydrogen atoms shown in Scheme 3 (see Fig. 3, and Fig. S8–S12† for all 7ACs). This theoretical calculation supports that the reaction of these 7-ACs would be initiated by hydrogen abstraction from the phenol group on their hydrolysed form. In fact, the smallest BDE values for hydrolysed coumarins C_334_ and C_314_ match with small stoichiometry values (*n* = 2) determined from the coumarin consumption at high concentration. Likewise, the larger BDE values for C_6H_, C_343_ and C_102_ agrees with higher stoichiometry (*n* > 4) related to the reaction with alkoxyl radicals (Table 1).

### 3.4 Analysis of products generated during the reaction with AAPH derived free radicals

To evaluate the oxidation mechanism that could involve the participation of HA on 7ACs free radical reaction, products analysis by UHPLC MS/MS were performed (Table 2).

**Table 2:** Coumarin oxidation products detected by UHPLC MS/MS

*R*_t_ (min)	[M + H]^+^*m*/*z*	Formula	MS2^a^	Assignments on Schemes 4 and 5
2.8	272.09	C_15_H_14_NO_4_	240.07 (–O_2_)	(2), R = H
3.85	242.11	C_6H_: C_15_H_15_NO_2_	214.05 (–CO)	(1), R = H
2.8	286.19	C_16_H_16_NO_4_	228.06 (–CH_3_, –3O), 254.07 (–O2)	(2), R = CH_3_
3.85	256.15	C_102_: C_16_H_17_NO_2_	228.02 (–CO), 241.04 (–CH_3_)	(1), R = CH_3_
2.98	344.18	C_17_H_14_NO_7_	312.15 (–O2)	(5), R = CH_3_
3.19	314.2	C_17_H_16_NO_5_	296.14 (–H_2_O), 256.13 (–CH_3_, –3O)	(4), R = CH_3_
3.64	284.2	C_334_: C_17_H_17_NO_3_	266.1 (–H_2_O)	(3), R = CH_3_
3.06	374.19	C_18_H_16_NO_8_	328.14 (–CH_2_, –2O)	(5), R = OC_2_H_5_
3.32	344.2	C_18_H_18_NO_6_	298.06 (–CH_2_, –2O)	(4), R = OC_2_H_5_
4.02	314.17	C_314_: C_18_H_19_NO_4_	268.08 (–H_2_O)	(3), R = OC_2_H_5_
2.73	316.1	C_16_H_12_NO_5_	298.12 (–H_2_O)	(7)^a^
3.09	286.12	C_343_: C_16_H_15_NO_4_	268.08 (–H_2_0)	(6)^a^

a MS2 fragmentation of coumarins and oxidation products are included in the ESI.

**Scheme 4:**
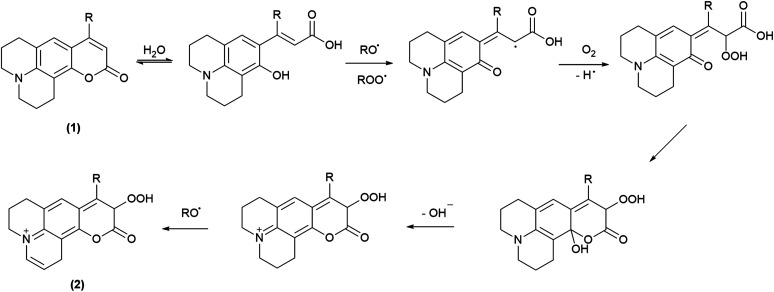
Propose oxidation steps involved on C_6H_ and C_102_.

**Scheme 5:**
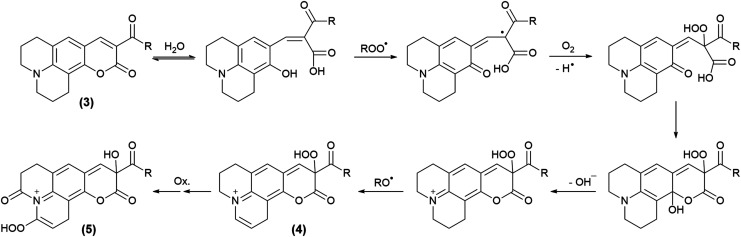
Propose oxidation steps involved on C_334_ and C_314_.

All the coumarin products detected can be proposed from an oxidation route derived from initial hydrogen abstraction from the phenolic OH group (C_H_OH, Table 1) with the lowest BDE (Schemes 4 and 5, and Scheme S1†). In fact, these structures agree with the fragmentation pattern observed on these products (Table S2 and S3, Scheme S2–S6†). Interestingly, all coumarins evaluated can be classified in two groups: (i) C_6H_, C_102_, and C_343_, which only one oxidation product is detected, and (ii) C_314_ and C_334_ that showed one oxidation product at short incubation times with formation of a second product at long incubation times. An important aspect is that the proposed intermediates and oxidation products are mainly hydroperoxides and endoperoxides. Moreover, the second product detected in the second group would require an additional hydrogen abstraction from the second labile group C–H_1_ (Table 1) that has been reported through photochemistry routes.^44–46^ In comparison with the kinetic data, it is seen that the second group includes the most reactive coumarins, which would involve peroxyl free radical mediated oxidation at short reaction times, whereas at long oxidation times the oxidation would be mediated by alkoxyl free radicals.

## 4. Conclusions

The kinetic information obtained from the reaction of 7ACs with AAPH derived free radicals strongly suggests remarkable antioxidant properties. This behaviour is attributed to participation of traces of hydroxycinnamic acid type compounds in equilibrium with 7ACs at neutral pH. Interestingly, amino group in these hydroxycinnamic acids would decrease BDE energy of the OH group allowing shifting their reaction towards alkoxyl or peroxyl radicals depending on the presence of substituents in the chemical structure able to favour hydrolysis and delocalize the spin density of the 7ACs free radical. That hypothesis is supported by theoretical results and our cumulative spectroscopic information, permitting to establish new undeveloped antioxidant behaviour for coumarins with potential uses on pharmacology.

## Conflicts of interest

The authors have no conflicts of interest to declare.

## Supplementary Material

RA-008-C7RA12000A-s001
