# Supplementary material for: Atypical antioxidant activity of non-phenolic amino-coumarins
Source: RSC Adv. 2018 Jan 9;8(4):1927–33. doi: 10.1039/c7ra12000a (PMC9077249; doi:10.1039/c7ra12000a)
Supplement: RA-008-C7RA12000A-s001 [file RA-008-C7RA12000A-s001.pdf]

Electronic Supplementary Material (ESI) for RSC Advances.
This journal is © The Royal Society of Chemistry 2017

Electronic Supplementary Information

Atypical Antioxidant Activity of Non-Phenolic Amino-Coumarins

Daniel Zúñiga-Núñez,[a] *Pablo Barrias,*[a] *Gloria Cárdenas-Jirón,*[a] *M. Soledad Ureta-Zañartu,*[a] *Camilo Lopez-Alarcón,*[b] *F. Eduardo Morán Vieyra,*[c] *Claudio D. Borsarelli,*[c] *Emilio I. Alarcon,*[d,e] *and Alexis Aspée.*[a,*]

[a]Facultad de Química y Biología, Universidad de Santiago de Chile, Casilla 40 Correo 33, Santiago, Chile.

[b]Departamento de Química Física, Facultad de Química, Pontificia Universidad Católica de Chile. C.P. 782 0436, Santiago, Chile.

[c]Instituto de Bionanotecnología del NOA (INBIONATEC). Universidad Nacional de Santiago del Estero (UNSE), CONICET. RN9, km 1125, CP4206 Santiago del Estero, Argentina

[d]Bio-nanomaterials Chemistry and Engineering Laboratory, Division of Cardiac Surgery, University of Ottawa Heart Institute, 40 Ruskin St., Ottawa, Ontario K1Y 4W7, Canada

[e]Department of Biochemistry, Microbiology, and Immunology, Faculty of Medicine, University of Ottawa, 451 Smyth Road, K1H 8M5 Ottawa, ON, Canada.

Corresponding author

Alexis Aspée, email: alexis.aspee@usach.cl

Table of contents

1. Evaluation of the free radical reaction of 7ACs by peroxyl free radicals..S3
2. Analysis multivariate for coumarin 314. ....S4
3. 7ACs consumption measurements...S6
4. Benesi-Hildebran analysis for o-coumaric acid-AAPH complex...S7
5. Function Fukui + for all coumarins S8
6. Hydrolysis of 7ACs measured in alkaline media. S9
7. Spin density and hydrolyzed form for all coumarins. S11
8. Oxidation route for C343...S16
9. Fragmentation of coumarins. S17
10. Fragmentation of all coumarin oxidation products...S20

1. Evaluation of the free radical reaction of 7ACs by peroxyl free radicals

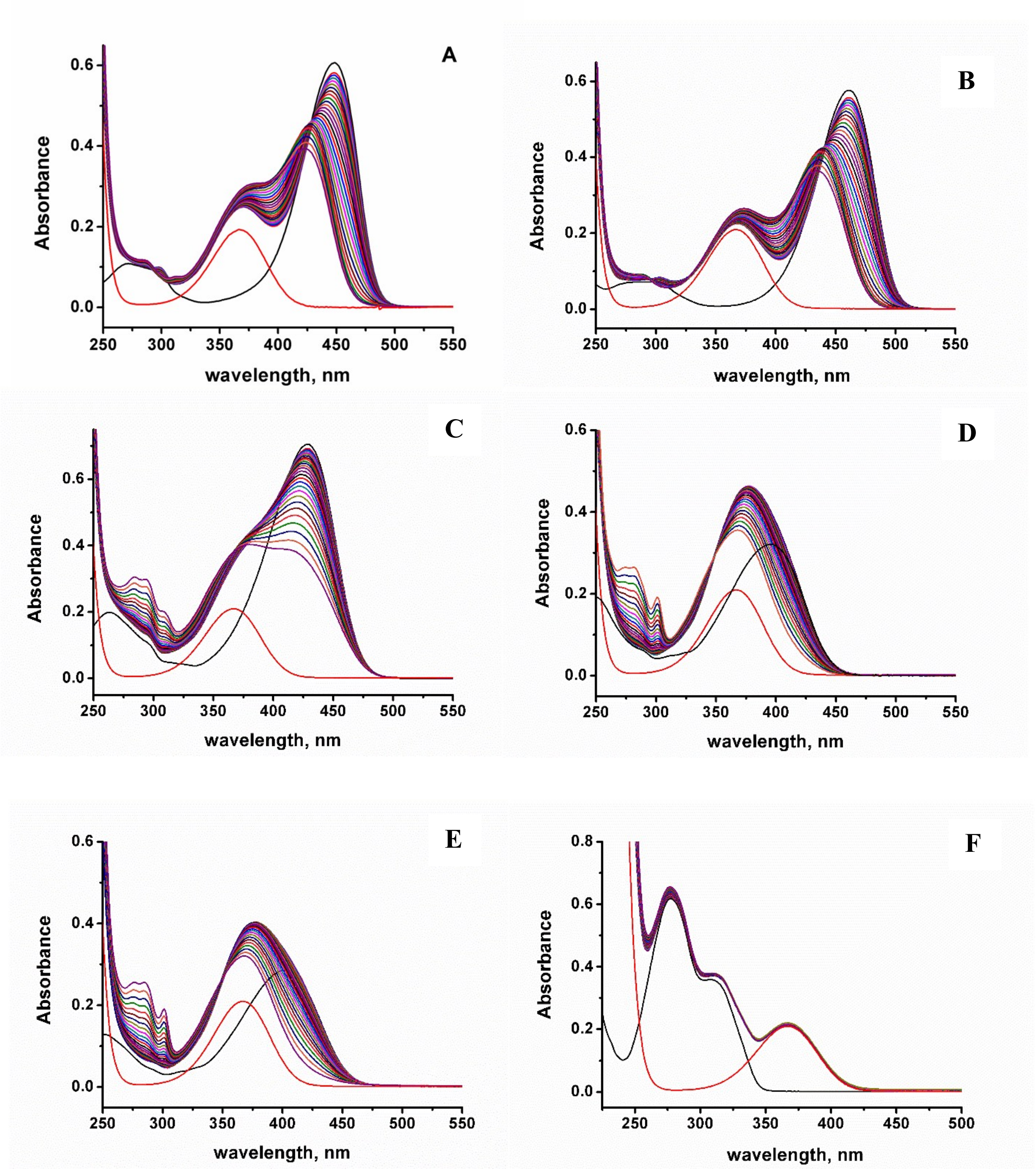

Figure S1. Consumption of 15 μM Coumarins (**—**) induced by incubation in AAPH 10 mM at 37 C, pH 7.0. 7ACs: C_{314} (A), C_{334} (B), C_{343} (C), C_{102} (D), C_{6H} (E) and C_{1} (F). UV-visible spectrum of 10 mM AAPH (**—**)

2. Analysis multivariate for coumarin 314.

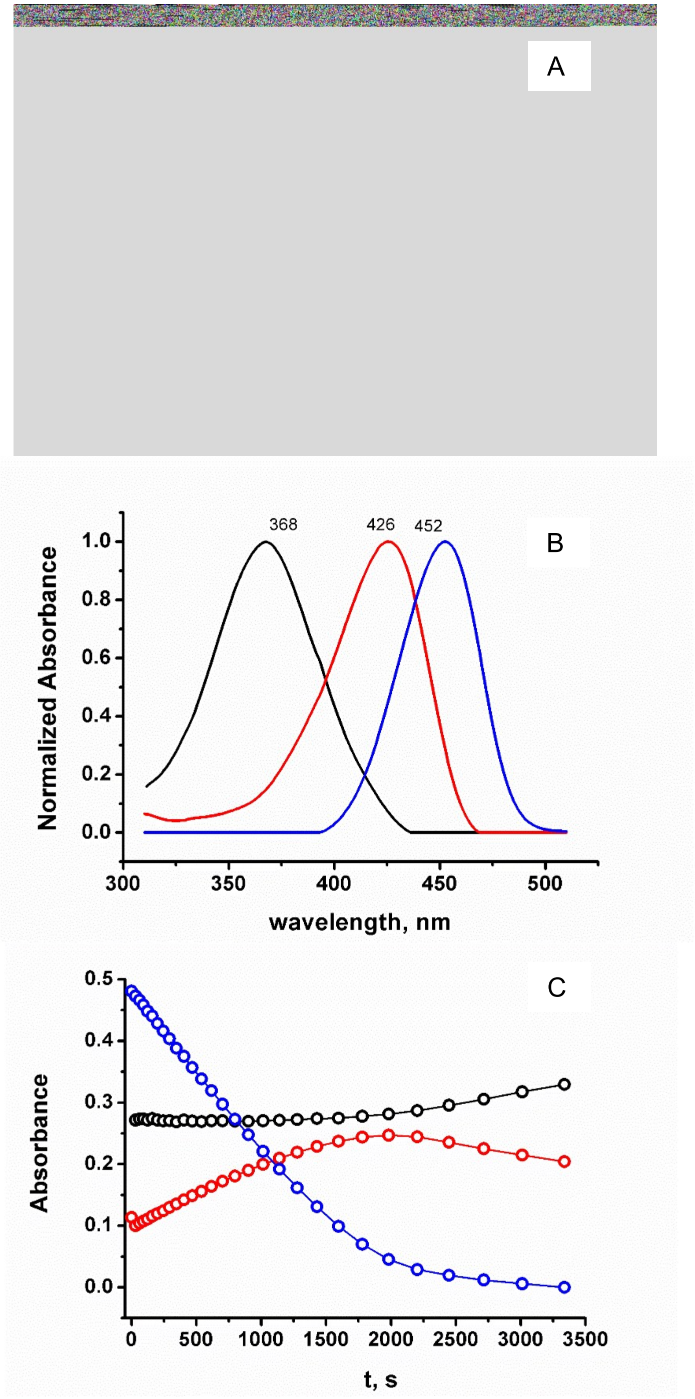

Figure S2. (A) Consumption of 15 μM C_{314} (▬) induced by incubation in AAPH 10 mM (▬) at 37 °C, pH 7.0. (B) Absorption spectrum and (C) kinetic profile of C_{314} (o), P1(o), P2 (o) by analysis multivariate.

Table S1. Recovered absorption band maxima (nm) obtained by MCR-ALS analysis with two or three components of the UV-Vis spectral evolution produced by peroxyl radical reaction with coumarin derivatives.

	**λ_1**	**λ_2**	**λ_3**	**r^2**	**LOF (%)**
**C_{6H}**	365	388		99.9639	0.7636
**C_{102}**	363	383		99.9176	0.8646
**C_{334}**	436 (372)*	466 (365)*		99.7147	**6.3416**
	367	437	465	99.9979	0.4631
**C_{343}**	371	430		99.8990	0.9291
**C_{314}**	424 (375)*	454 (364)*		99.7635	**5.8626**
	367	426	454	99.9976	0.4933

*band shoulder or overlapped maximum. LOF (%): Lack of fit (optimization parameter). The spectral analysis is shown in the ESI.

3. 7ACs consumption measurements

.

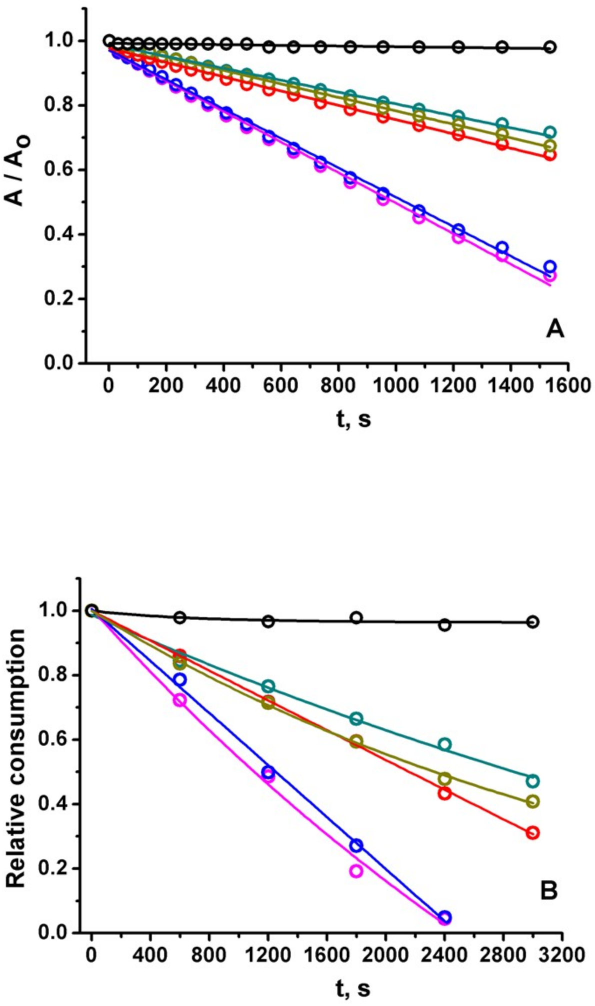

Figure S3. (A) Consumption of 15 μM 7ACs and C_1 induced by incubation on AAPH 10 mM at 37 C, pH 7.0, estimated by fluorescence measurements and by (B) UHPLC MS/MS. 7ACs: C_{6H} (o), C_{334} (o), C_{343} (o), C_{314} (o), and C_{102} (o) and C_1 (o).

4. Benesi-Hildebran analysis for o-coumaric acid-AAPH complex

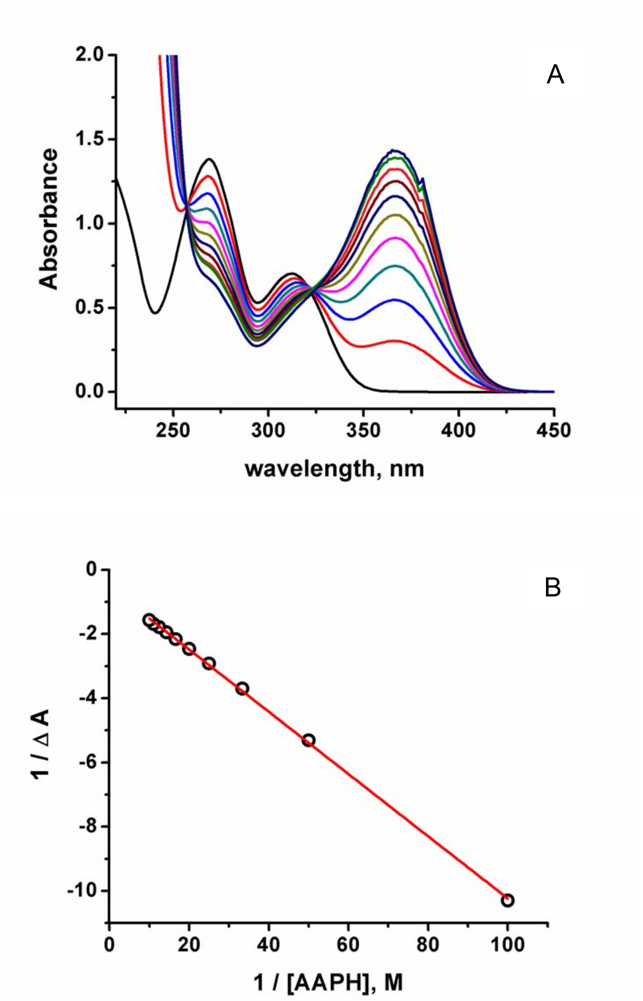

Figure S4. (A) UV-visible absorbance spectra of 90 μM o-coumaric acid measured at different AAPH concentrations at 4º C. (B) Evaluation of the Benessi-Hildebran plot for o-coumarin acid absorbance decrease band at 325 nm against AAPH concentration.

5. Function Fukui + for all coumarins

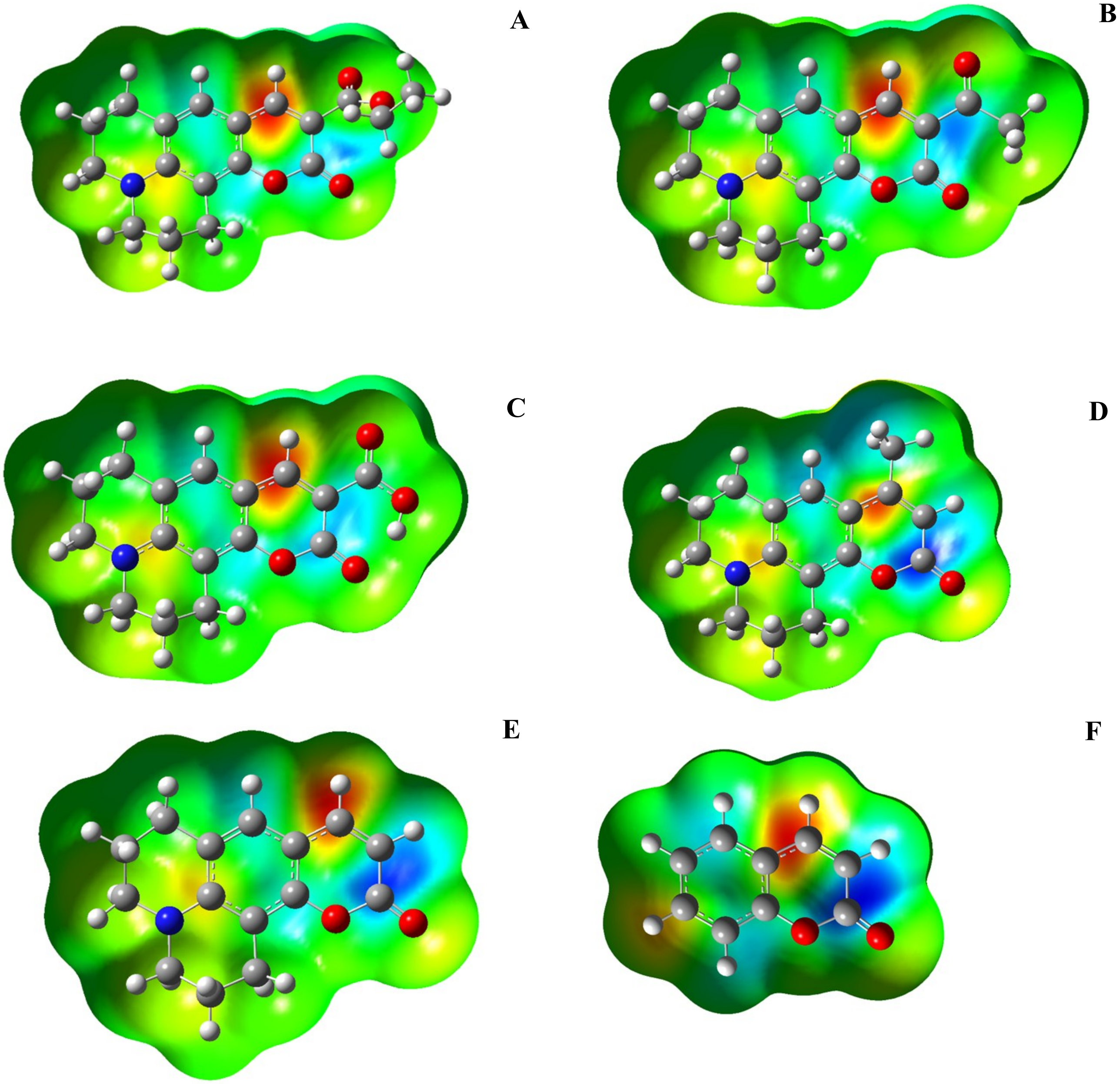

Figure S5. Isosurphase function of Fukui+ for all coumarins. computed using B3LYP/6-31G (d,p) and a conductor-like polarizable continuum model (C-PCM) with the standard parameters form water. 7ACs: C_{314} (A), C_{334} (B), C_{343} (C), C_{102} (D), C_{6H} (E) and C_1 (F).

6. Hydrolysis of 7ACs measured in alkaline media.

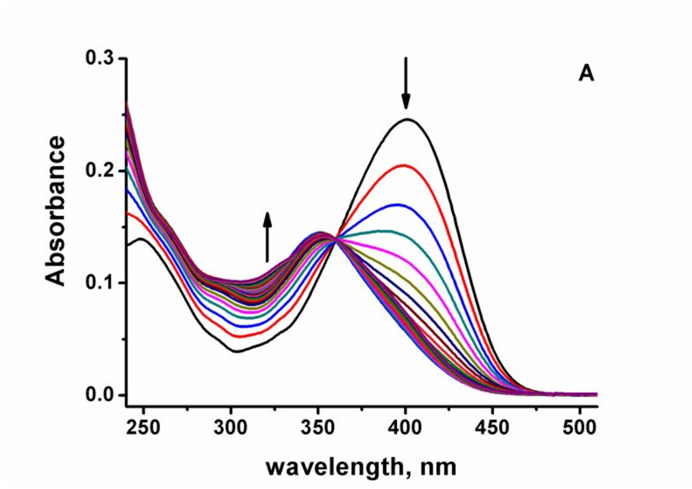

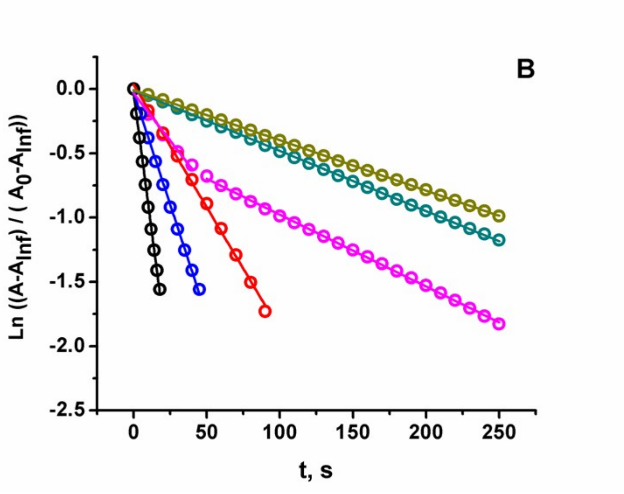

Figure S6. (A) UV-visible absorbance spectra of 15 μM C_{6H} measured at different incubation times in 1M NaOH, and pseudo first order kinetic consumption of 7ACs evaluated from absorbance measurements at the maximum absorbance bands (B). 7ACs: C_{6H} (o), C_{334} (o), C_{343} (o), C_{314} (o), C_{102} (o) and C_1 (o). Determined apparent hydrolysis rate constants of 0.0191, 0.0348, 0.0047, 0.0039, 0.0860 s^{-1} were calculated for C_{6H}, C_{334}, C_{343}, C_{102} and C_1, respectively. Initial rate constant for C_{314} (0.0148 s^{-1}) represent hydrolysis to C_{334} with a further hydrolysis to cinnamic acid derivative (0.0056 s^{-1}).

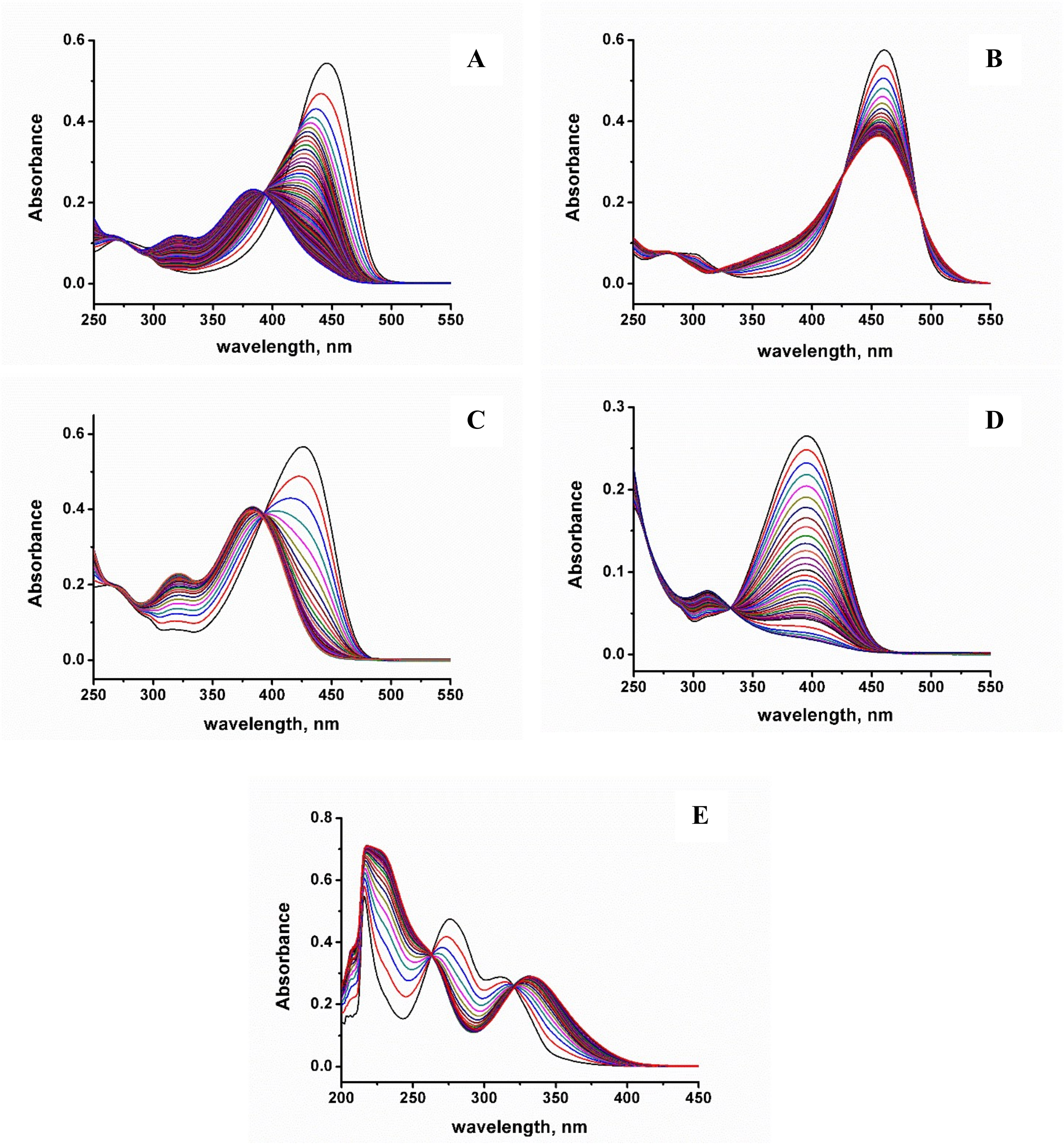

Figure S7. UV-visible absorbance spectra of 15 μM 7ACs measured at different incubation times in 1M NaOH and C_1 at different incubation times in buffer pH 12. 7ACs: 7ACs: C_{314} (A), C_{334} (B), C_{343} (C), C_{102} (D), and C_1 (E).

7. **Spin density and hydrolyzed form for all coumarins.**

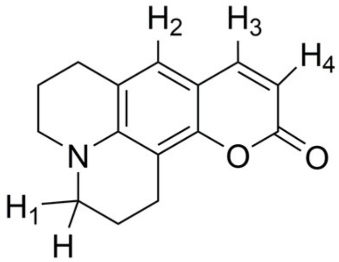

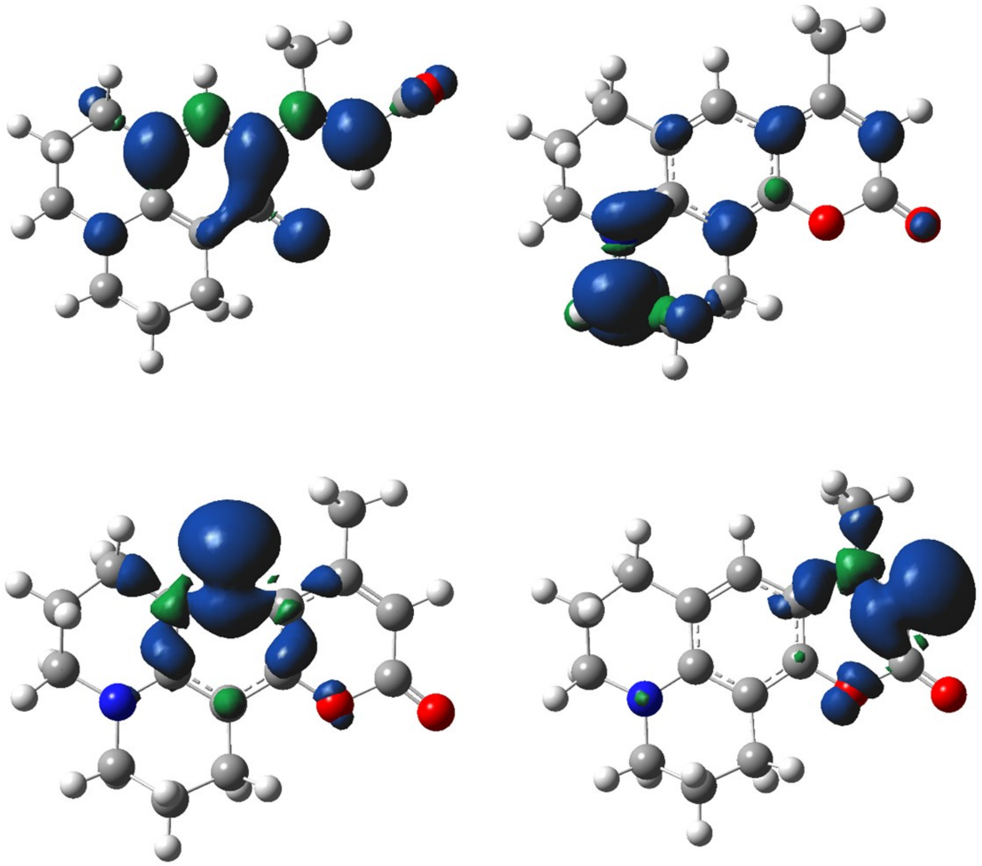

Figure S8. (A) Spin density of coumarin 6H hydrolyzed free radical (top, left) and spin density of coumarin 102 cycle form by free radical of abstraction of H1 (top, right), H2 (bottom, left) and H3 (bottom, right) at an isosurface value of 0.002, computed using B3LYP/6-31G (d,p) and a conductor-like polarizable continuum model (C-PCM) with the standard parameters form water.

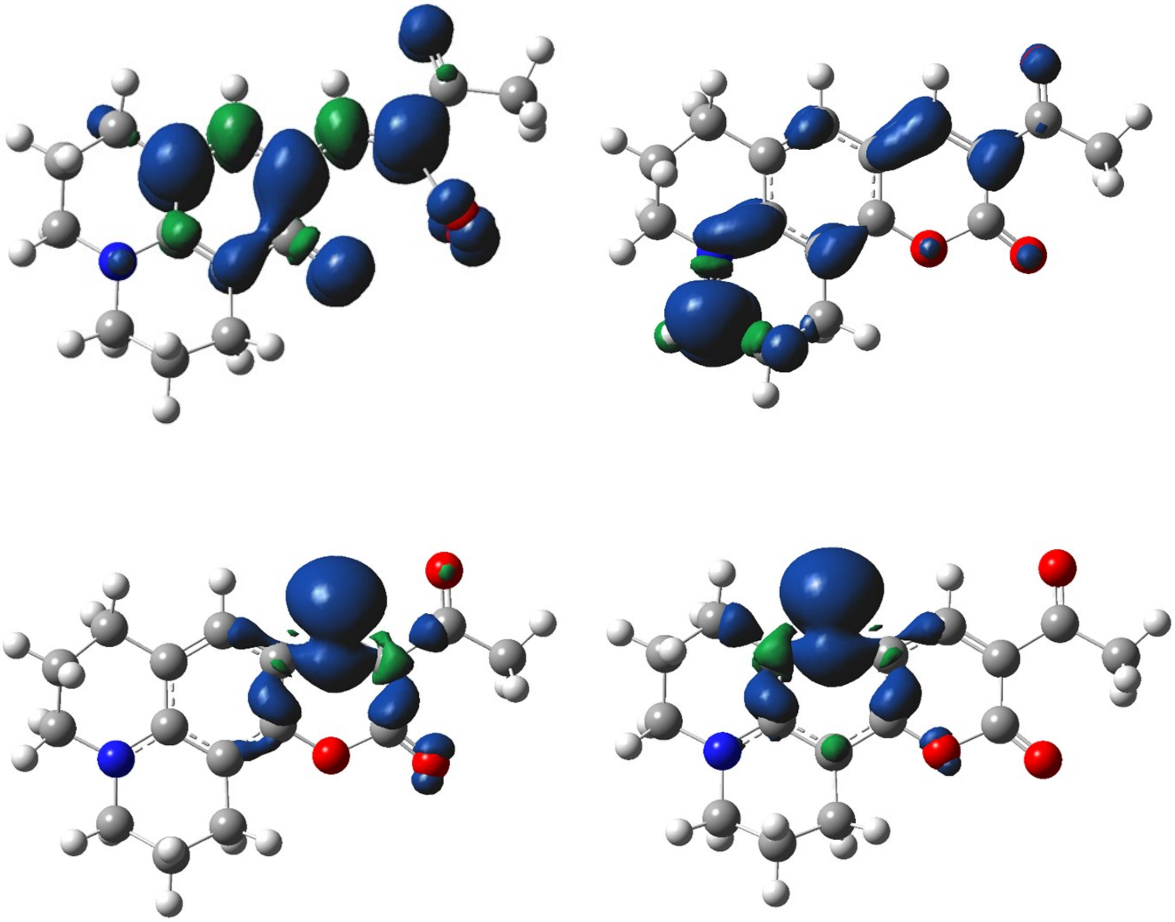

Figure S9. (B) Spin density of coumarin 334 hydrolyzed free radical (top, left) and spin density of coumarin 334 cycle form by free radical of abstraction of H1 (top, right), H2 (bottom, left) and H3 (bottom, right) at an isosurface value of 0.002, computed using B3LYP/6-31G (d,p) and a conductor-like polarizable continuum model (C-PCM) with the standard parameters form water.

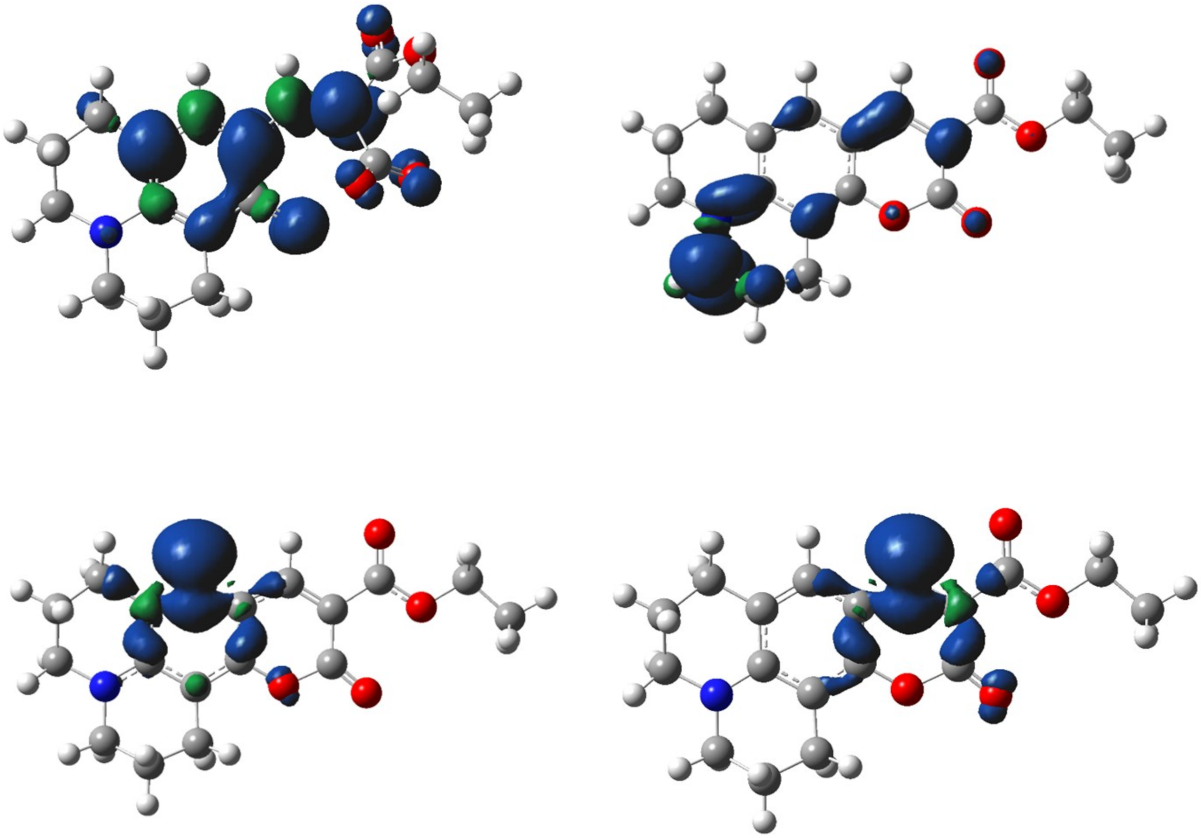

Figure S10. (C) Spin density of coumarin 314 hydrolyzed free radical (top, left) and spin density of coumarin 314 cycle form by free radical of abstraction of H1 (top, right), H2 (bottom, left) and H3 (bottom, right) at an isosurface value of 0.002, computed using B3LYP/6-31G (d,p) and a conductor-like polarizable continuum model (C-PCM) with the standard parameters form water.

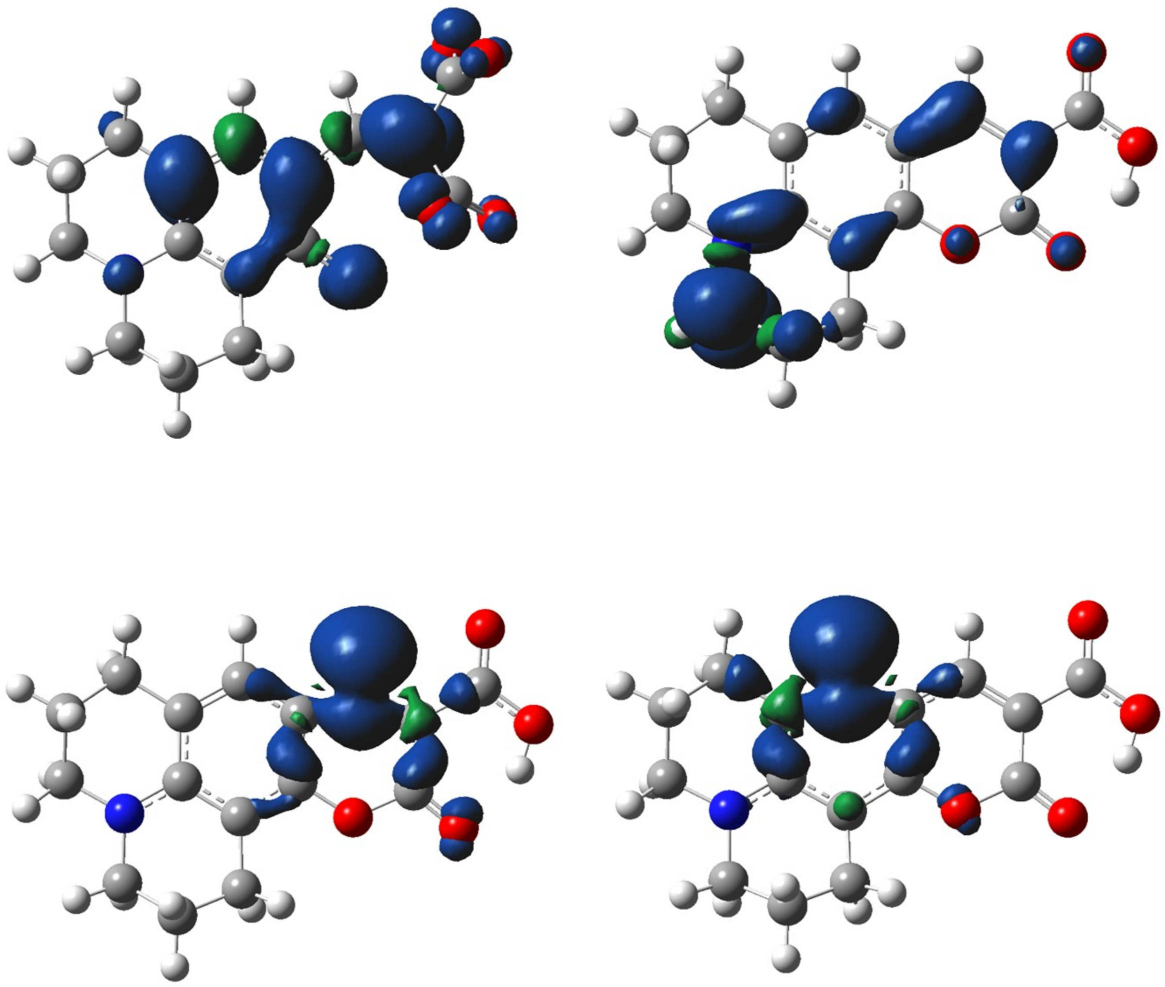

Figure S11. (D) Spin density of coumarin 343 hydrolyzed free radical (top, left) and spin density of coumarin 314 cycle form by free radical of abstraction of H1 (top, right), H2 (bottom, left) and H3 (bottom, right) at an isosurface value of 0.002, computed using B3LYP/6-31G (d,p) and a conductor-like polarizable continuum model (C-PCM) with the standard parameters form water.

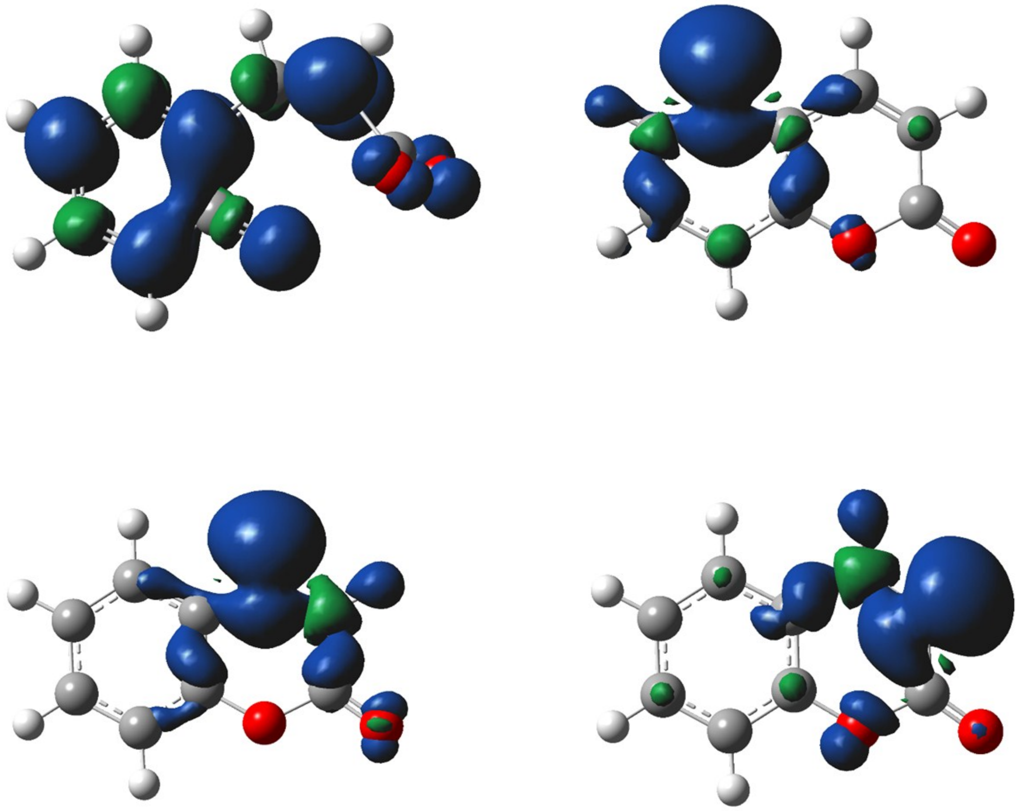

Figure S12. (E) Spin density of coumarin (C1) hydrolyzed free radical (top, left) and spin density of coumarin cycle form by free radical of abstraction of H2 (top, right), H3 (bottom, left) and H4 (bottom, right) at an isosurface value of 0.002, computed using B3LYP/6-31G (d,p) and a conductor-like polarizable continuum model (C-PCM) with the standard parameters form water.

8. Oxidation route for C343.

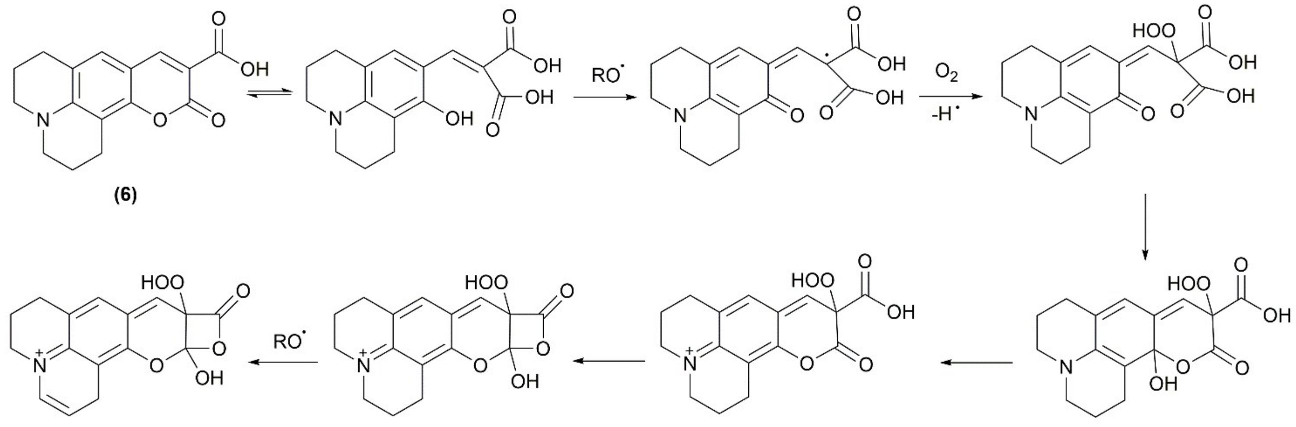

Scheme S1. Oxidation route considering an initial hydrogen abstraction from the phenolic OH group for the hydrolysed C_{343} by incubation of AAPH at 37°C.

9. Fragmentation of coumarins.

Table S2. Mass and Fragmentation of 7-dialkyl-aminocoumarins measured by UHPLC-MS/MS.

Coumarin	$[M^{+}H]^{+}$	MS2	MS3
6H	244.11	214.05	186.06
102	256.15	228.02<br>241.04	200.08
334	284.2	266.1	-
314	314.17	268.08	-
343	286.12	268.08	-

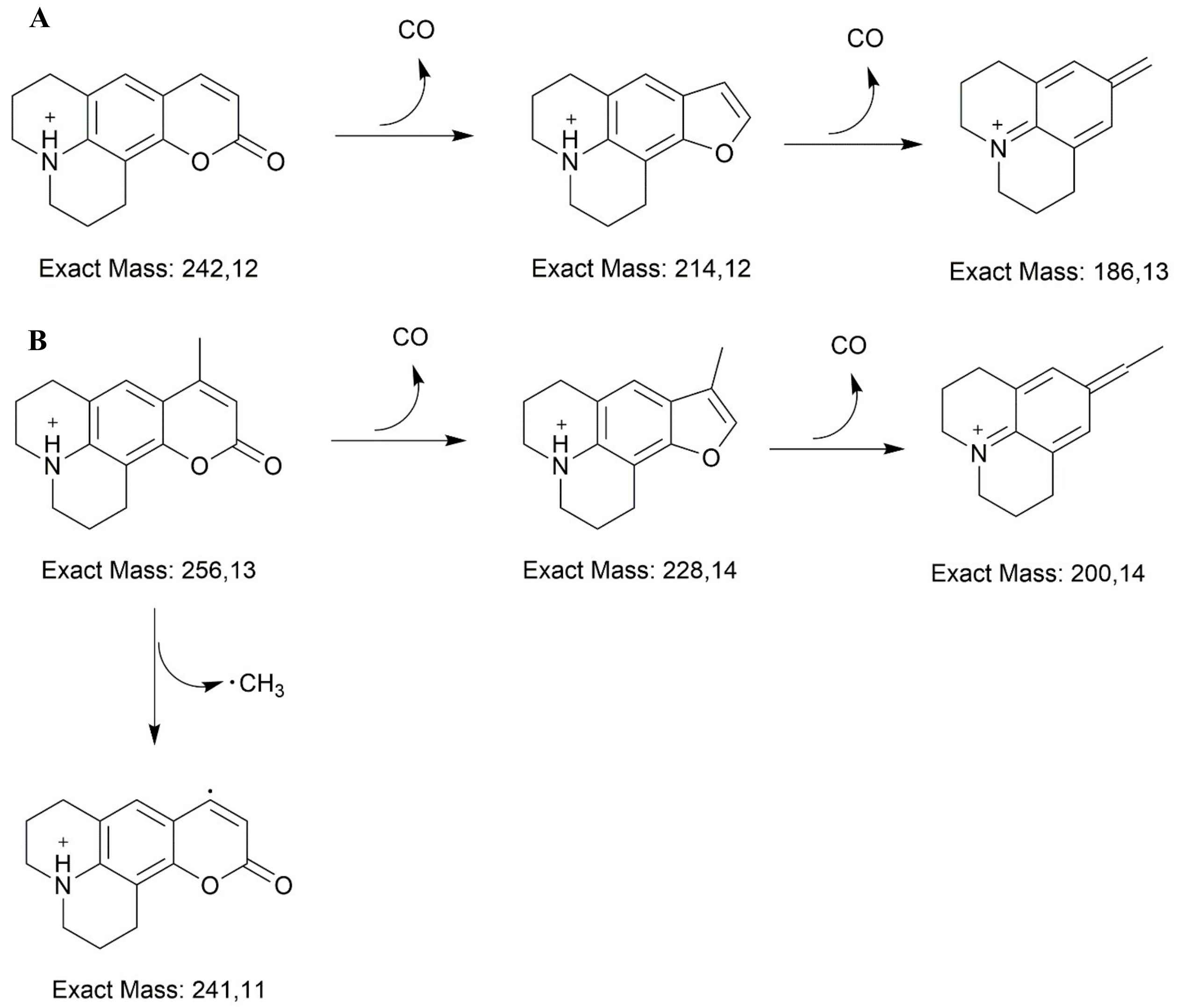

Scheme S2. Fragmentation of coumarins by UHPLC / MSMS. (A) C_{6H}, (B) C_{102}.

C

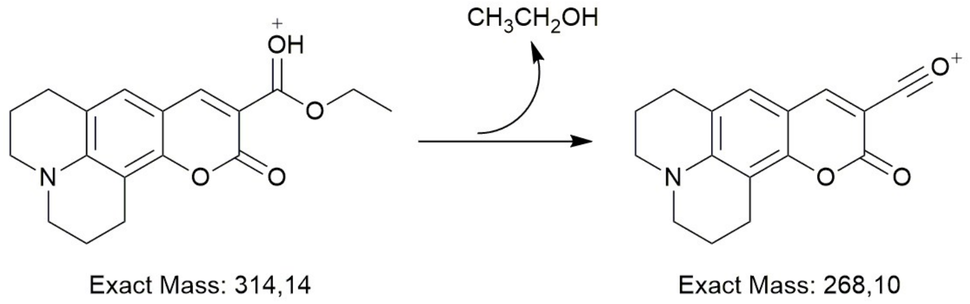

D

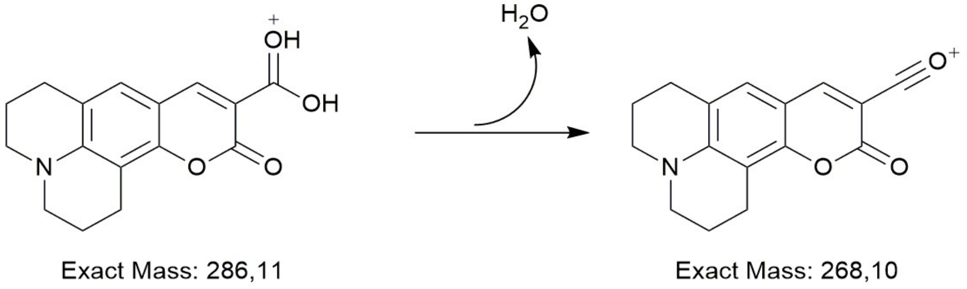

E

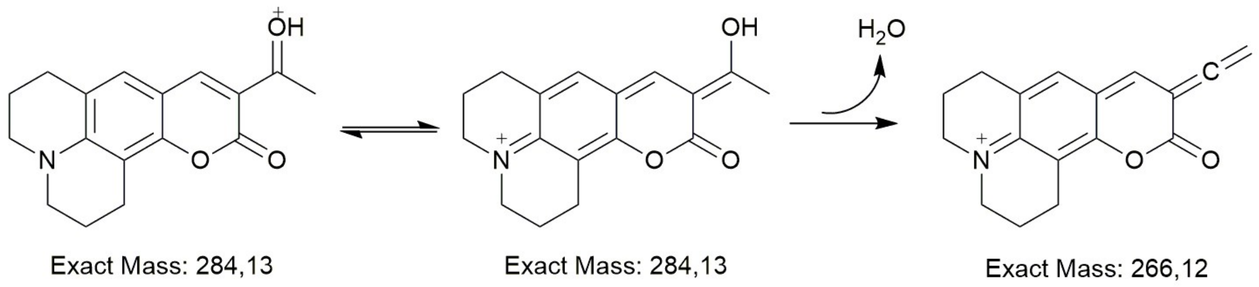

Scheme S3. Fragmentation of coumarins by UHPLC / MSMS. (C) C_{314}, (D) C_{343}, (E) C_{334}.

10. Fragmentation of all coumarin oxidation products.

Table S3. Mass and Fragmentation of 7-dialkyl-aminocoumarins oxidation products detected by UHPLC-MS/MS.

**Coumarin**	**[M+H] +**	**MS2**	**MS3**
6H	272.09	240.07	212.08
102	286.19	254.07/228.06	226.08
334	314.2<br>344.19	296.14 / 256,13<br>312.15	265.15<br>-
314	344.2<br>374.19	298.06<br>328.14	211.0<br>-
343	316.1	298.12	211.06 / 242.16 / 270.19

A

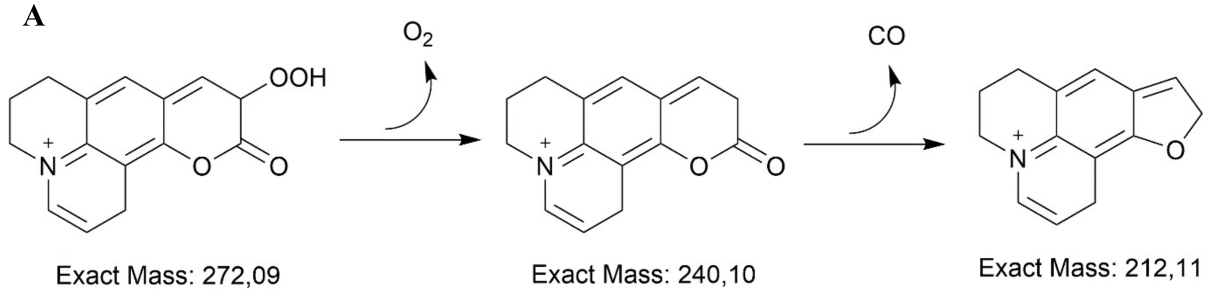

B

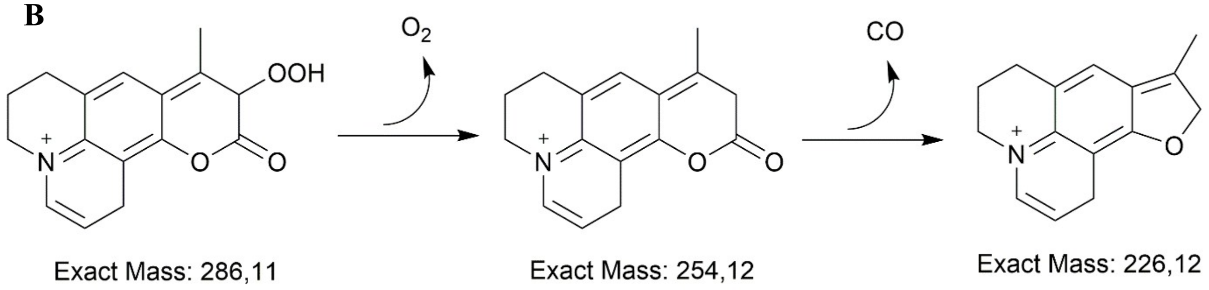

Scheme S4. Fragmentation of coumarin oxidation products from reaction with incubated AAPH 10 mM at 37 °C, pH 7.0 by UHPLC / MSMS. (A) C_{6H}, (B) C_{102}.

C

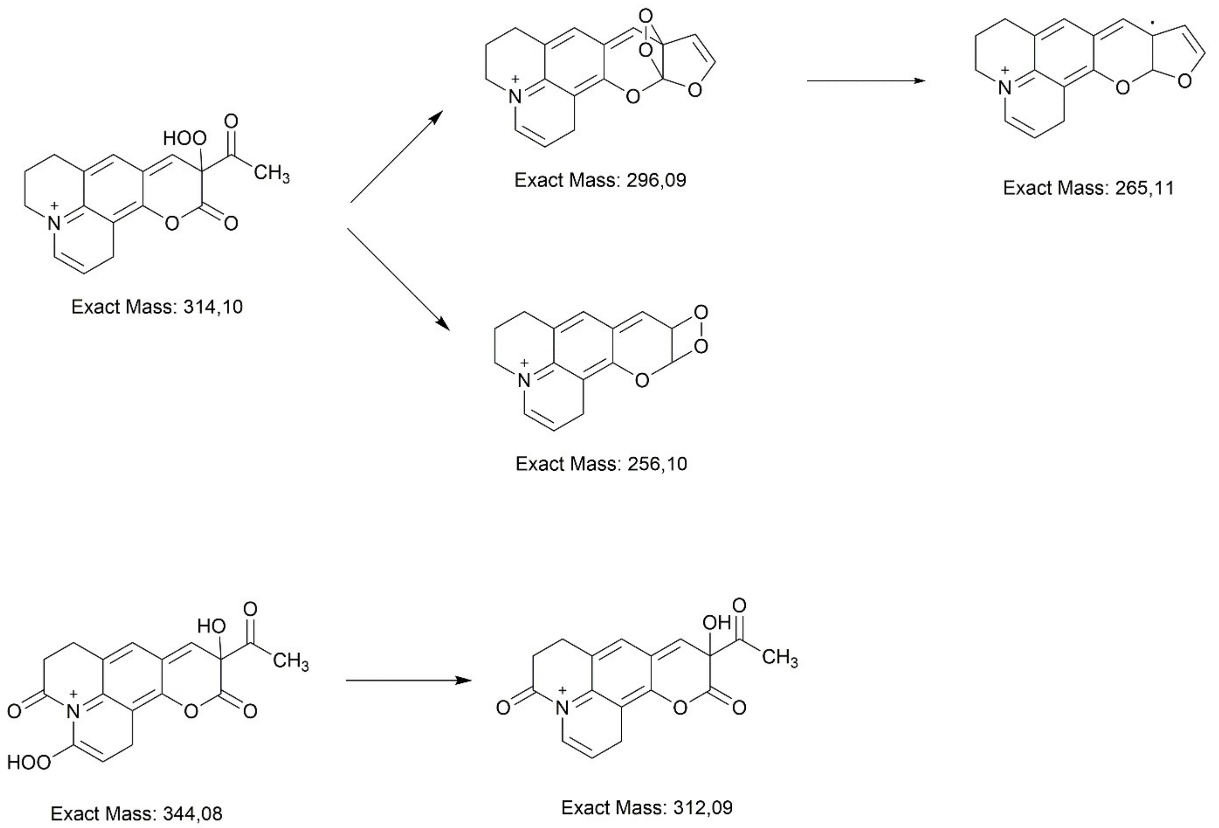

Scheme S5. Fragmentation of coumarin oxidation products from reaction with incubated AAPH 10 mM at 37 °C, pH 7.0 by UHPLC / MSMS. (C) C_{334}.

D

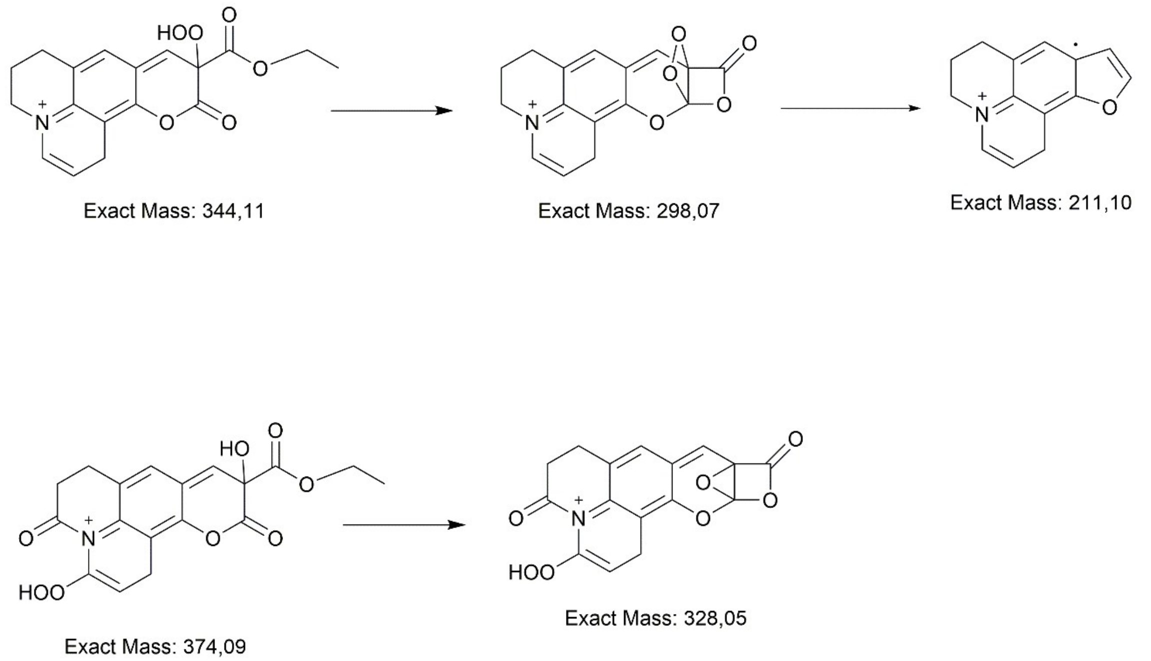

E

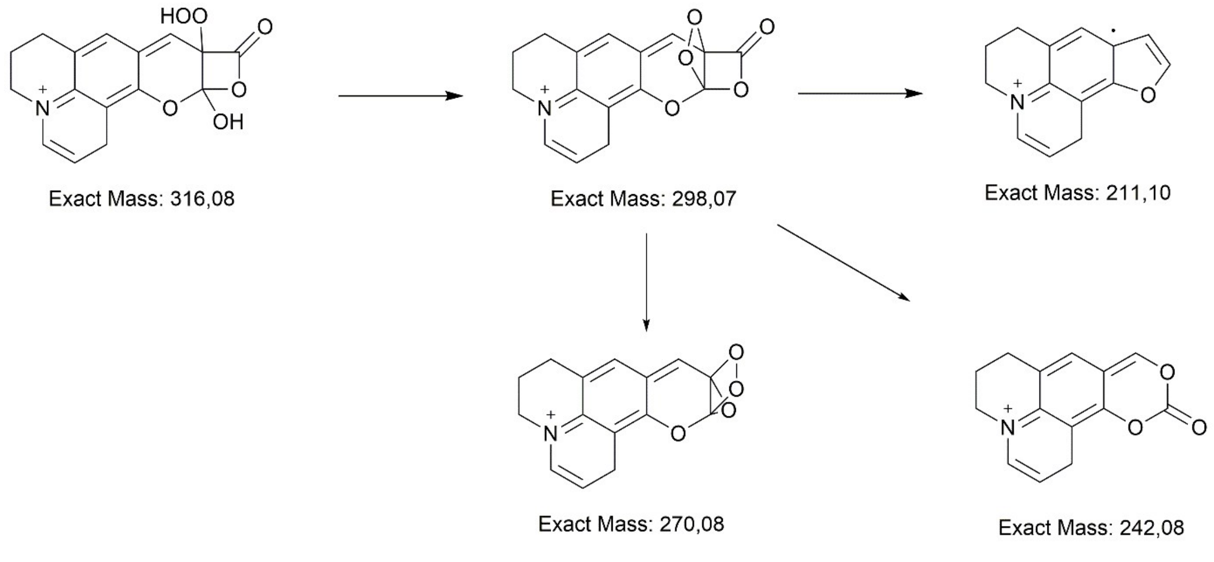

Scheme S6. Fragmentation of coumarin oxidation products from reaction with incubated AAPH 10 mM at 37 °C, pH 7.0 by UHPLC / MSMS. (D) C_{314}, (E) C_{343}.
